# Alphabets, musical notes, and nucleotide sequences: some commonalities and significant differences

**DOI:** 10.3325/cmj.2020.61.574

**Published:** 2020-12

**Authors:** Charles H. Calisher

**Affiliations:** calisher@cybersafe.net

To this sometimes eccentric series of opinions and ideas I add here what may appear to be three apparently unrelated subjects: alphabets, music notes, and genes. By comparing and contrasting them, I will try to demonstrate that biological evolution may be a never-ending process.

**Alphabets**: There are more than 7000 languages spoken in the world at this time. A few are the most widely spoken, a sad few are the least spoken. Most languages are based on alphabets (the first two letters of the Greek alphabet are “alpha” and “beta”), which is a standardized set of basic written symbols or graphemes (called letters) that represent the phonemes (distinct units of sound) of certain spoken languages. Each letter in the English language has an upper- and lower-case form and originated around the seventh century from Latin script. Originally published in 1884 by Oxford University Press (1), the Second Edition (1989) of the 20-volume Oxford English Dictionary contains full entries for 171 476 words in current use and 47 156 obsolete words. Because various languages may contain different numbers of letters in their alphabets, languages can fluctuate in their possible word constructs. For example, Hebrew has 22 letters, Portuguese 23, Greek 24, English, French, German, and Klingon 26, Arabic 28, Finnish 29, Croatian and Spanish 30, Russian 33, Slovak and Indian 46. The Chinese language does not have an alphabet *per se*. Rather, the Chinese use 3000-6000 pictograms, pictures, or symbols that convey meaning through their resemblance to physical objects. France has one official language required by the French constitution to protect it from being “contaminated” through the incursion of other languages, such as English, but it has many unofficial languages

Interestingly, most languages evolve by addition, replacement, and invention, but modern life necessitates the utility of incorporating new words, ie, cosmonaut, ecoanxiety, Latinx, pansexual, bombogenesis, and many more. As human life changes, so do languages. One might call all this “language evolution.” It occurs relatively slowly but continually.

**Musical notes**: These are another group of fascinating descriptors. In about 500 B.C.E., Pythagoras of Samos (2), whose thoughts later influenced the philosophies of Plato, and Aristotle, and, through them, Western philosophy (3), began investigating musical notes. Among other things, he is credited with discovering that a string exactly half the length of another will play a pitch that is exactly an octave higher when struck or plucked. Split a string into thirds and you raise the pitch an octave and a fifth. Split it further and the pitch is proportionally higher. Delightful sounds all seem somehow related to mathematics, do they not?

Anyway, there are 12 notes in 7 letters (A, A sharp, B, C, C sharp, D, D sharp, E, F, F sharp, G, G sharp) and it has been calculated that there can be 82.5 × 10^17^ combinations of such notes. Therefore, it is unlikely that we will run out of music any time soon. Compound these permutations with pitch, meter, flatted, sharped, major, minor, beats, repetition (think of Ludwig Van Beethoven's 5th Symphony in C Minor or Maurice Ravel’s “Bolero”), etc. (I do not know what “etc.” means. This is more complex than I can understand; I am in way over my head in musical waters) and we can be certain that acceptable music, rather than what is being invented today, will be available for “discovery” for a very long time. In a way, music also evolves along with society but it is less a regular progression and more the punctuated equilibrium ascribed to biological evolution by Stephen Jay Gould (1941-2002) (4), paleontologist, evolutionary biologist, historian of science, and fan of baseball until the end of his life.

**Nucleotide sequences** (5): There are four major classes of biochemical compounds: nucleic acids, carbohydrates, proteins, and lipids. Nucleic acids are polynucleotide chain structures that include ribonucleic acid (RNA) and DNA (DNA). Both types of nucleic acids contain the elements carbon, hydrogen, oxygen, nitrogen, and phosphorus. Nucleotides are defined as compounds composed of a phosphate group, a sugar (deoxyribose in DNA and ribose in RNA), and one of four nitrogen-containing bases. In DNA, the bases are adenine, thymine, guanine, and cytosine, while RNA has the base uracil instead of thymine, and other three bases are the same as those in DNA.

If appropriately positioned so that a ribosome can effectively access them, any three nucleotides are considered a codon, the 3-nucleotide structure that codes for an amino acid, the compounds that constitute proteins. Given that the genetic code is read in such triplets and that there are four possible bases that can reside in each position, the number of possible codons is 64 (4 × 4 × 4). However, there are only 20 recognized amino acids (Ala = alanine, Arg = arginine, Asn = asparagine, Asp = asparagine, Cys = cysteine, Gln = glutamine, Glu = glutamate, Gly = glycine, His = histidine, Ile = isoleucine, Leu = leucine, Lys = lysine, Met = methionine, Phe = phenylalanine, Pro = proline, Ser = serine, Thr = threonine, Trp = tryptophan, Tyr = tyrosine, and Val = valine). It is well established that three codons (UAA, UAG, and UGA) function as “stop” codons which terminate signals during translation (decoding). These tell the cell when and where a polypeptide is complete; how they “know” to do this is unknown to me. Including these three, there are 23 triplets. Thus, each amino acid can be specified by more than a single codon, as shown below for RNA.

UUU Phe UCU Ser UAU Tyr UGU Cys

UUC Phe UCC Ser UAC Tyr UGC Cys

UUA Leu UCA Ser UAA (stop) UGA (stop)

UUG Leu UCG Ser UAG (stop) UGG Trp

CUU Leu CCU Pro CAU His CGU Arg

CUC Leu CCC Pro CAC His CGC Arg

CUA Leu CCA Pro CAA Gln CGA Arg

CUG Leu CCG Pro CAG Gln CGG Arg

AUU Ile ACU Thr AAU Asn AGU Ser

AUC Ile ACC Thr AAC Asn AGC Ser

AUA Ile ACA Thr AAA Lys AGA Arg

AUG* Met ACG Thr AAG Lys AGG Arg

GUU Val GCU Ala GAU Asp GGU Gly

GUC Val GCC Ala GAC Asp GGC Gly

GUA Val GCA Ala GAA Glu GGA Gly

GUG Val GCG Ala GAG Glu GGG Gly

^*^Also functions as a start codon.

Because most DNA is double-stranded, it has a built-in proof-reading mechanism. That is, if a replication error is made in one of the strands, the two strands do not “fit” together and the product is less likely to be successful. Alternatively, if an error is made during RNA synthesis, such an error can yield a successful variant. That is not always the case, of course, but it is possible. Error-prone RNAs, rather than DNA as found in cells, can actually serve as the genomes for many of the viruses that we know and (not) love. Thus, variation in viral genomes is a fact of life. Success of a virus variant is a matter of fitness, its capacity to produce infectious progeny in a given environment. The new virus may have biological characteristics (asymptomatic hosts, higher rate of infectivity, greater pathogenicity in some hosts, host organ specificity, aerosol transmission, greater environmental stability, etc.) that provide it with increased success, such as is possessed by the SARS-CoV-2 virus that causes coronavirus disease 2019.

What then are the commonalities and dissimilarities among alphabets, musical notes, and nucleotide sequences that form the title of this column? Alphabets in any language are limited by long-standing tradition. Most linguists are comfortable with whatever letters they have available in their language of interest. Anyway, the 171 476 words in the Oxford English Dictionary that the 26 letters comprise and which are in current use (or at least available) for those of us who speak English should be enough for anyone. Relatively gradual additions of newly invented words in English typically do not produce a series of great leaps forward so, thankfully, we will not be competing with the Chinese alphabet any time soon, or ever, I hope. I must admit that, from time to time, I enjoy browsing through the Oxford dictionary. I always find words I had never before seen but sometimes find a new favorite word, for example “tenebrific.”

Musical notes allow remarkable complexities, even though they comprise only 12 notes (in 7 letters), allowing 82.5 × 10^17^ note combinations. Obviously, we will not soon come to the end of new music. Still, it was astonishing geniuses, such as Wolfgang Mozart, Ludwig van Beethoven, and James Brown who put the notes in such order as to produce unforgettable music. The notes already were available to others but the music they produced was remarkably inventive, but neither essential nor naturally occurring.

It is, however, the specific codon placements that produce genetic variability and allow for biological diversity. Where this will end, if it does end, is unknown. Make one small change (substitution) in a viral non-structural protein and the phenotype of that virus can be profoundly changed.

Unlike alphabet letters and musical notes, random substitutions (“accidents,” “mutants”) may, for example, change a virus with low infectivity and low pathogenicity to one with high infectivity and high pathogenicity. Remarkable! For words, random additions (strange/strangle), subtractions (strange/range), or replacements (insert/insect) can make huge differences. Changes in single musical notes are even worse, perhaps mistakenly suggesting the need to place a call to a piano tuner.

Most natural virus mutations are random and appear to be harmless, even if functional, at least for a time. However, whereas most mutations are unfit and do not replicate further, they continuously and endlessly occur and, eventually, when one is successful, a biologically suitable entity is created, “a piece of bad news wrapped up in protein,” as Nobel Prize Laureate Peter B. Medawar classically described a virus. Obviously, it is unknown where all these successful mutations, including mutations of mutations in perpetuity, eventually will lead us on the Road of Evolution (6). It might be a good idea to continue such investigations, however. If there is any evidence for the existence of God, this may be it.
